# Elementary Quantitative Analysis

**Published:** 1878-10-20

**Authors:** 


ELEMENTARY QUANTITATIVE ANALYLIS, by Alexander Classen, Professor in the Royal Polytechnic School Aixlachapelle. Translated with additions, by Edgar F. Smith A. M., Ph. D. Assistant in Anolytical Chemestry, etc., in University of Pensylvania, with thirty-six illustrations. Henry C. Lea 1878.

The above work is used as a text-book in the leading laboratories, and polytechnic schools in Germany, and presents to the student of Chemestry, a compact and highly useful manual of quantitative anslysis.

